# Prevalence and Correlates of Hedonic Hunger Among Youth Attending a Primary Health Center in South India: A Cross-Sectional Study

**DOI:** 10.7759/cureus.100785

**Published:** 2026-01-04

**Authors:** Kasula Nimilitha, Vijay Kishore A, Vignesh D, Nalukurthi Midhun Teja, Rajeev Aravindakshan, Arti Gupta

**Affiliations:** 1 Department of Community and Family Medicine, All India Institute of Medical Sciences Mangalagiri, Mangalagiri, IND

**Keywords:** hedonic hunger, india, obesity prevention, power of food scale, primary healthcare, youth

## Abstract

Background and objective

Hedonic hunger, defined by eating driven by pleasure rather than physiological need, contributes significantly to obesity and metabolic disorders. Limited data exist on hedonic hunger prevalence among Indian youth, particularly in primary healthcare settings. The present study aimed to estimate the prevalence of hedonic hunger and its sociodemographic and lifestyle correlates among youth aged 15-29 years in a South Indian primary healthcare setting.

Methods

A cross-sectional study was conducted among 241 youth attending a Primary Health Center in Guntur, Andhra Pradesh. Hedonic hunger was measured using the Power of Food Scale (PFS). Sociodemographic data, lifestyle factors, dietary behaviors, and anthropometric measurements were collected. High hedonic hunger was defined as a PFS score greater than 34. Associations were analyzed using chi-square tests and multivariable logistic regression.

Results

All 241 participants provided valid PFS scores, resulting in a 100% response rate. The mean age was 24.0 ± 4.6 years, with 67.6% being female. The mean PFS score was 27.80 ± 9.93. The prevalence of high hedonic hunger was 25.3%, with a 95% confidence interval (CI) of 20.2% to 31.2%. High hedonic hunger was significantly more prevalent among males than females (38.5% vs. 19.0%, p = 0.002). Multivariable analysis revealed that the influence of advertisements (adjusted odds ratio (OR) = 6.00), family income below ₹15,000 (adjusted OR = 5.33), and smoking habit (adjusted OR = 10.85) were significant predictors of high hedonic hunger.

Conclusions

High hedonic hunger affected one-quarter of youth in this South Indian primary healthcare setting, with notable gender disparities. Strong associations with modifiable lifestyle factors suggest key targets for intervention. These findings highlight the need to incorporate hedonic hunger assessment into routine primary care and to develop targeted behavioral interventions for youth.

## Introduction

The global obesity epidemic has reached alarming proportions, with India experiencing a rapid nutrition transition marked by increasing consumption of energy-dense, nutrient-poor foods among young populations [1,2]. Traditional models of eating behavior have focused primarily on homeostatic mechanisms that regulate food intake to maintain energy balance [3]. However, emerging evidence suggests that hedonic pathways, namely the brain’s reward and pleasure systems, play an equally important role in driving food consumption, particularly in environments with abundant palatable food options [4].

Hedonic hunger, described as eating driven by the anticipation of pleasure rather than physiological need, represents a critical factor in understanding contemporary eating behaviors [5]. Unlike homeostatic hunger, which arises from caloric requirements, hedonic hunger is triggered by the sight, smell, or thought of palatable foods and can override signals of satiety [6]. This pleasure-driven eating pattern has been linked to greater consumption of high-calorie foods, weight gain, and metabolic dysfunction [7,8]. The Power of Food Scale (PFS), developed by Lowe et al., provides a validated measure of hedonic hunger by assessing sensitivity to food reward and the psychological impact of living in a food-abundant environment [9]. Studies from Western populations have demonstrated that higher PFS scores are associated with overeating, weight gain, and difficulty with weight management [10]. Hedonic hunger is higher among individuals who are young, physically inactive, consume nighttime snacks, and follow weight loss diets [11].

Youth aged 15-29 years constitute 27.2% of the Indian population and are disproportionately exposed to aggressive food marketing, evolving food environments, and lifestyle transitions [12]. This demographic faces unique challenges, including academic stress, peer influences, increased autonomy over food choices, and exposure to Western dietary patterns through media and globalization [13]. Primary healthcare centers act as key points of contact with this population, offering opportunities for early identification and intervention. Previous Indian studies have identified concerning trends in eating behaviors among youth, including higher consumption of processed foods, irregular meal patterns, and emotional eating [14,15]. Understanding the prevalence and correlates of hedonic hunger in Indian youth is essential for developing culturally appropriate interventions and informing public health strategies. The present study examines hedonic hunger prevalence and its associations with sociodemographic and lifestyle factors among youth attending the Centre for Rural Health, All India Institute of Medical Sciences (AIIMS), Guntur district, South India.

## Materials and methods

Study design and setting

A cross-sectional study was conducted at the outpatient department of the Centre for Rural Health, AIIMS, PHC Nutakki in Guntur, Andhra Pradesh, South India. The study was conducted over two months in 2025, after obtaining approval from the Institutional Ethics Committee of AIIMS, Mangalagiri (Certificate No. AIIMS/MG/IEC/2024-25/47, September 19, 2024). All study procedures adhered to the ethical principles outlined in the Declaration of Helsinki.

Participants

The study population comprised 78 males and 163 females aged 15-29 years attending the PHC outpatient department. Inclusion criteria were youth aged 15-29 years, residence in the PHC catchment area for at least six months, and provision of written informed consent, with assent obtained for adolescents. Exclusion criteria included serious acute illness, pregnancy, known psychiatric disorders, and intellectual disabilities that could impair questionnaire completion.

Sample size and sampling

Sample size was calculated using the formula n = 4pq/d², with an expected hedonic hunger prevalence of 68.1% based on a Turkish study [16], 6% absolute precision, and a 95% confidence interval (CI), yielding a required sample of 241 participants. A pilot study was conducted with 16 participants to test the feasibility of the questionnaire and data collection procedures before the main study. Systematic random sampling was employed to recruit eligible participants attending the PHC outpatient department until the target sample size was reached.

Data collection

Sociodemographic and Lifestyle Assessment

Informed written consent was obtained from the study participants. For adolescent participants, informed written consent was obtained from the parent or caregiver, and written assent was obtained from the adolescent. A structured questionnaire collected information on age, gender, education, occupation, family income, family type, and lifestyle factors, including tobacco use, alcohol consumption, and physical activity patterns. Lawshe's technique was used to assess the content validity ratio (CVR). A panel of 10 experts from community and family medicine, general medicine, nursing, lay persons, and teachers analyzed each item for relevance. The subject experts rated each item on a three-point scale with options: a) not necessary, b) useful but not essential, and c) essential. The CVR was determined using the formula for each item. (N = the total number of experts and n = the number of experts who have selected option c for each item). Items with a CVR score greater than 0.80 were retained.

Hedonic Hunger Assessment

Hedonic hunger was measured using the 15-item Power of Food Scale (PFS) developed by Lowe et al. [6]. Permission to use the PFS was obtained from Drexel University, Philadelphia, PA. The PFS assesses sensitivity to food reward across three domains: food availability, food present, and food tasted. Each item is rated on a 5-point Likert scale from 1 ("I do not agree") to 5 ("I strongly agree"). Total scores range from 15 to 75, with higher scores indicating greater hedonic hunger. High hedonic hunger was defined as a total PFS score greater than 34, consistent with previous research [16].

Dietary and Media Behaviors

Participants were assessed for junk food consumption patterns, watching television while eating [17], and susceptibility to food advertisements through structured questions. Junk food was defined as processed foods high in sugar, salt, or fat, including chips, baked goods, and aerated drinks.

Anthropometric Measurements

Weight was measured using calibrated SECA 213 digital scales (accuracy ± 0.1 kg) and height using SECA 813 stadiometers (accuracy ± 0.1 cm). BMI was calculated as weight (kg)/height (m²). BMI categories were defined using WHO Asian cutoffs: underweight (<18.5), normal (18.5-22.9), overweight (23.0-27.4), and obese (≥27.5) [18].

Statistical analysis

Data were analyzed using Python (version 3.12) with pandas, numpy, scipy, and statsmodels libraries. Continuous variables were presented as mean ± standard deviation (SD), and categorical variables as frequencies and percentages. Hedonic hunger was dichotomized into high hedonic hunger vs. low hedonic hunger. Bivariate associations between hedonic hunger categories and participant characteristics were examined using chi-square tests for categorical variables or Fisher's exact test when expected cell counts were less than 5. Multivariable logistic regression was performed using maximum likelihood estimation to identify independent predictors of high hedonic hunger. Variables were selected for inclusion based on bivariate associations (p<0.20) and clinical relevance. Results are presented as adjusted odds ratios (OR) with 95% CI. Model fit was assessed using McFadden's Pseudo R², Akaike Information Criterion (AIC), and likelihood ratio test. Statistical significance was set at p<0.05 (two-tailed).

## Results

Participant characteristics

All 241 eligible participants provided complete PFS responses and were included in the final analysis (100% response rate). The mean age was 24.0 ± 4.6 years (range: 15-29). The majority were female (163, 67.6%), had completed high school or higher education (218, 90.4%), and lived in nuclear families (131, 54.4%). Most participants were housewives (111, 46.1%) or not currently employed (66, 27.4%). The majority of participants had family incomes predominantly in the lower-to-middle range, with 186 (77.2%) earning <₹25,000 per month (Table 1).

**Table 1:** Sociodemographic and lifestyle characteristics of study participants (N = 241)

Variable	Category	N (%)
Age groups, years	15-19	51 (21.2)
	20-24	66 (27.4)
	25-29	124 (51.5)
Gender	Female	163 (67.6)
	Male	78 (32.4)
Educational level	Primary/middle school	23 (9.5)
	High school	70 (29.0)
	Intermediate	74 (30.7)
	Graduate+	74 (30.7)
Occupation	Housewife	111 (46.1)
	Not working/student	66 (27.4)
	Private job	24 (10)
	Daily labor	13 (5.4)
	Others	27 (11.2)
Family income, *₹*	<15,000	40 (16.6)
	₹15,000-25,000	146 (60.6)
	₹25,000-50,000	42 (17.4)
	>₹50,000	13 (5.4)
Family type	Nuclear	131 (54.4)
	Joint/extended	108 (44.8)
	Single	2(0.8)
Current smoking status	Yes	9 (3.7)
	No	232 (96.3)
Alcohol use	Yes	16 (6.6)
	No	225 (93.4)
Junk food consumption	Yes	197 (81.7)
	No	44 (18.3)
Eating while watching TV	Yes	127 (52.7)
	No	112 (46.5)
Advertisement influence	Yes	74 (30.7)
	No	166 (68.9)
BMI categories, kg/m²	Underweight (<18.5)	22 (9.1)
	Normal (18.5-22.9)	93 (38.6)
	Overweight (23.0-27.4)	92 (38.2)
	Obese (≥27.5)	34 (14.1)

Regarding lifestyle factors, nine (3.7%) reported current tobacco use, 16 (6.6%) consumed alcohol, and 197 (81.7%) regularly consumed junk food. Watching television while eating was reported by 127 (52.7%) of participants, with 74 (30.7%) indicating that food advertisements influenced their eating behavior.

Hedonic hunger prevalence and distribution

The mean PFS total score was 27.80 ± 9.93 (median: 25.0, interquartile range (IQR): 20-35, range: 14-64). The distribution was approximately normal with a slight right skew. High hedonic hunger (PFS >34) was observed in 61 participants, yielding a prevalence of 25.3% (95% CI: 20.2%-31.2%) (Table 2).

**Table 2:** Prevalence and of hedonic hunger distribution of PFS scores (N = 241) PFS: Power of Food Scale; SD: standard deviation; IQR: interquartile range; CI: confidence interval; HH: hedonic hunger

Measure	Statistic	Value
PFS total score	Valid responses	241/241 (100.0%)
	Mean ± SD	27.80 ± 9.93
	Median (IQR)	25.0 (20-35)
	Range	14-64
PFS subscale (number of items)		
Food available (6)	Scores	12.56 ± 4.87
Food present (7)	Mean ± SD	6.17 ± 3.16
Food tasted (2)		9.07 ± 2.85
High hedonic hunger (PFS >34)	Prevalence	61/241 (25.3%)
	95% CI	20.2-31.2%
By gender	High HH - females	31/163 (19.0)
	High HH - males	30/78 (38.5)
By age group	High HH - 15-19 years	12/51 (23.5)
	High HH - 20-24 years	20/66 (30.3)
	High HH - 25-29 years	29/124 (23.4)

Association with sociodemographic factors

High hedonic hunger prevalence differed significantly by gender, with males showing higher rates compared to females (38.5% vs. 19%, χ² = 9.546, p = 0.002). Significant associations were also found with family income (p<0.001), with particularly high prevalence among the lowest income group (<₹15,000: 57.5%). No significant associations were found with age groups, education level, or BMI categories (Table 3).

**Table 3:** Association between sociodemographic factors and hedonic hunger (N = 241) BMI: body mass index; HH: hedonic hunger

Variable	Category	High HH, n (%)	Low HH, n (%)	Chi-square	P-value
Age group, years	15-19	12 (23.5%)	39 (76.5%)	χ² = 1.198	0.549
	20-24	20 (30.3%)	46 (69.7%)		
	25-29	29 (23.4%)	95 (76.6%)		
Gender	Female	31 (19.0%)	132 (81.0%)	χ² = 9.546	0.002
	Male	30 (38.5%)	48 (61.5%)		
Educational level	Graduate+	16 (21.6%)	58 (78.4%)	χ² = 2.771	0.428
	High school	22 (31.4%)	48 (68.6%)		
	Intermediate	16 (21.6%)	58 (78.4%)		
	Primary/middle school	7 (30.4%)	16 (69.6%)		
Occupation	Daily labor	4 (30.8%)	9 (69.2%)	χ² = 6.105	0.191
	Housewife	21 (18.9%)	90 (81.1%)		
	Not working/student	23 (34.8%)	43 (65.2%)		
	Others	6 (22.2%)	21 (77.8%)		
	Private Job	7 (29.2%)	17 (70.8%)		
Family income, *₹*	<15,000	23 (57.5%)	17 (42.5%)	χ² = 32.571	<0.001
	₹15,000-25,000	21 (14.4%)	125 (85.6%)		
	₹25,000-50,000	12 (28.6%)	30 (71.4%)		
	>₹50,000	5 (38.5%)	8 (61.5%)		
BMI category, kg/m^2^	Normal (18.5-22.9)	24 (25.8%)	69 (74.2%)	χ² = 3.924	0.270
	Obese (≥27.5)	8 (23.5%)	26 (76.5%)		
	Overweight (23.0-27.4)	27 (29.3%)	65 (70.7%)		
	Underweight (<18.5)	2 (9.1%)	20 (90.9%)		

Association with lifestyle factors

Strong associations were observed between hedonic hunger and several lifestyle factors. Advertisement influence showed the strongest association, with 42 (56.8%) of those influenced by ads having high hedonic hunger compared to 19 (11.4%) of those not influenced (χ² = 53.069, p<0.001). A significant association was observed between eating while watching television and high hedonic hunger scores, with prevalence rates of 48( 37.8%) among television watchers compared to 13 (11.6%) among non-watchers (p<0.001).

Junk food consumption showed a borderline association with high hedonic hunger (χ² = 3.162, p = 0.075), with prevalence of 55 (27.9%) among regular consumers vs. six (13.6%) among non-consumers. This finding approached but did not achieve statistical significance. Neither alcohol consumption nor junk food frequency patterns demonstrated significant associations with hedonic hunger (Table 4).

**Table 4:** Association between lifestyle factors and hedonic hunger (N = 241) Missing data: TV Eating (n=2), Advertisement Influence (n=1), Junk Food Frequency (n=43) HH: hedonic hunger

Variable	Category	High HH, n (%)	Low HH, n (%)	Chi-square	P-value
Current smoking status	Yes	7 (77.8%)	2 (22.2%)	χ² = 10.883	<0.001
	No	54 (23.3%)	178 (76.7%)		
Alcohol use	Yes	4 (25.0%)	12 (75.0%)	χ² = 0.000	1.000
	No	57 (25.3%)	168 (74.7%)		
Junk food consumption	Yes	55 (27.9%)	142 (72.1%)	χ² = 3.162	0.075
	No	6 (13.6%)	38 (86.4%)		
Junk food frequency	Once a day	2 (25.0%)	6 (75.0%)	χ² = 0.427	0.808
	Once a week	23 (24.7%)	70 (75.3%)		
	Once a month	28 (28.9%)	69 (71.1%)		
Watching television while eating	Yes	48 (37.8%)	79 (62.2%)	χ² = 20.117	<0.001
	No	13 (11.6%)	99 (88.4%)		
Advertisement influence	Yes	42 (56.8%)	32 (43.2%)	χ² = 53.069	<0.001
	No	19 (11.4%)	147 (88.6%)		

Multivariable analysis

In multivariable analysis, three variables emerged as significant independent predictors of high hedonic hunger: low family income (<₹15,000/month) showed 5.3-fold increased odds (adjusted OR = 5.33, 95% CI: 2.16-13.13, p<0.001), current smoking demonstrated 10.9-fold increased odds (adjusted OR = 10.85, 95% CI: 1.48-79.59, p=0.019), and advertisement influence showed six-fold increased odds (adjusted OR = 6.00, 95% CI: 2.50-14.39, p<0.001) (Table 5).

**Table 5:** Multivariable logistic regression analysis for high hedonic hunger (N = 241) OR: odds ratio; CI: confidence interval

Variable	Adjusted OR	95% CI	P-value
Age (per year)	1.06	0.95-1.18	0.326
Gender (male vs. female)	1.43	0.66-3.11	0.363
Education (graduate+ vs. others)	1.09	0.47-2.53	0.849
Occupation (student/not working vs. others)	2.85	0.93-8.75	0.067
Family income (	5.33	2.16-13.13	<0.001
Current smoking status (yes vs. no)	10.85	1.48-79.59	0.019
BMI (per kg/m²)	1.06	0.95-1.17	0.314
Junk food consumption (yes vs. no)	1.99	0.63-6.26	0.239
Eating while watching TV (yes vs. no)	1.88	0.72-4.94	0.198
Advertisement influence (yes vs. no)	6.00	2.50-14.39	<0.001

## Discussion

This study provides the first comprehensive assessment of the prevalence of hedonic hunger among youth in an Indian primary healthcare setting, using a validated instrument. Our finding of a 25.3% prevalence of high hedonic hunger is substantially lower than the 68.1% reported in the Turkish validation study [16]. However, it falls within the range reported in other populations using similar cutoffs [18]. This difference may reflect cultural variations in food environments, eating patterns, and the expression of food reward sensitivity.

Gender disparities

The significantly higher prevalence of high hedonic hunger among males (38.5% vs. 19.0%) is a notable finding that contrasts with some Western studies reporting a female predominance [19]. This gender difference may reflect cultural factors specific to the Indian context, including different food socialization patterns, varying degrees of food autonomy, and distinct social pressures related to eating behaviors between males and females [20]. In traditional Indian families, males may have greater food autonomy and exposure to external food environments, potentially increasing their susceptibility to hedonic eating patterns. Additionally, cultural expectations around male eating behaviors may be more permissive of pleasure-driven food consumption [21]. Understanding these gender-specific patterns is crucial for developing targeted interventions.

Socioeconomic factors

The strong association between low family income and hedonic hunger is particularly striking: 55.0% of those earning <₹15,000 exhibit high hedonic hunger, compared with much lower rates in higher-income groups. This finding suggests that economic constraints may paradoxically increase vulnerability to hedonic eating, possibly through mechanisms such as stress-induced eating, limited access to high-quality foods, which increases reward sensitivity, or differential exposure to food marketing targeting lower-income populations.

Lifestyle determinants

The strong association between advertising influence and hedonic hunger (adjusted OR = 6.00) among the most frequently reported relationships in the hedonic hunger literature. This finding is particularly concerning given the aggressive food marketing targeting Indian youth through digital platforms and traditional media [22]. Food advertisements are specifically designed to activate reward pathways and create desire for palatable foods, and our results suggest that youth who report being influenced by these advertisements have dramatically elevated risk for hedonic eating patterns [23].

Watching television while eating can be a predictor, consistent with research demonstrating that screen-based activities can disrupt attention to satiety cues and promote mindless eating [24]. The association may also reflect the synergistic effect of simultaneous exposure to food advertisements during television viewing, creating environmental cues that trigger hedonic responses [25]. The association between junk food consumption and hedonic hunger suggests a potentially bidirectional relationship where hedonic hunger drives consumption of palatable processed foods, which in turn may sensitize reward pathways and increase hedonic hunger [26]. This finding is particularly relevant given the rapid expansion of processed food markets in India and the increasing availability of Western-style fast foods [27].

Mental health and coping mechanisms

An important consideration not assessed in this study is the bidirectional relationship between hedonic hunger and mental health. Emerging evidence suggests that hedonic hunger is closely linked to psychological distress, with individuals experiencing anxiety, depression, or chronic stress often exhibiting heightened reward-driven eating behaviors. In this context, hedonic hunger may serve as a maladaptive coping mechanism, wherein palatable food consumption provides temporary relief from negative emotional states through activation of brain reward circuits [28]. Economic hardship induces chronic stress about financial security, while students and unemployed youth face academic pressure, uncertain career prospects, and identity formation challenges. These populations may be particularly susceptible to using hedonic eating as an accessible means of emotional regulation.

Future research should incorporate comprehensive mental health assessments to elucidate the complex interplay between psychological factors, socioeconomic stressors, and hedonic hunger. Understanding whether hedonic hunger mediates the relationship between mental health and obesity, or whether psychological interventions can reduce hedonic eating, would inform integrated prevention strategies addressing both mental and metabolic health in at-risk youth.

Clinical and public health implications

These findings have several important implications for primary healthcare practice. First, the substantial prevalence of high hedonic hunger suggests that routine assessment of eating motivations could enhance understanding of patients' relationships with food beyond traditional nutritional counselling. The PFS could be integrated into routine youth health assessments, particularly for those presenting with weight concerns or concerning eating patterns. Second, the identification of modifiable risk factors provides clear targets for intervention. Programs addressing media literacy, mindful eating practices, and reducing susceptibility to food advertisements could potentially reduce hedonic hunger and its associated health risks. Primary healthcare providers are well-positioned to deliver such interventions given their trusted relationships with patients and families. Third, the strong male predominance identified suggests the need for gender-tailored approaches that account for differential vulnerabilities and social contexts. Male-focused interventions might emphasize awareness of advertisement manipulation tactics and the development of critical thinking skills around food marketing.

The exceptionally high odds ratios observed for advertisement influence and eating while watching TV suggest that interventions targeting these factors could have a substantial population-level impact. Media literacy programs teaching youth to recognize and resist food marketing techniques could be particularly valuable.

Study strengths and limitations

The comprehensive assessment of sociodemographic and lifestyle factors allows for a thorough characterization of correlates. The primary healthcare setting provides validity for the target population. However, a few limitations should be acknowledged. The cross-sectional design precludes causal inferences about the direction of observed associations. The study was conducted in a single primary healthcare center, potentially limiting generalizability to other settings or regions of India. Self-reported measures of dietary behaviors may be subject to social desirability bias, though the use of validated instruments helps mitigate this concern. The sample size calculation was based on prevalence estimates from Western populations. A more conservative approach, assuming 50% prevalence, would have yielded a larger sample size. However, our achieved sample provided adequate power for detecting the primary associations of interest. The PFS was developed and validated in Western populations, and while we used a version adapted for similar cultural contexts, further validation in Indian populations would strengthen confidence in our findings. The sample size, while adequate for primary analyses, may have limited power for some subgroup analyses.

Future research directions

Future research should examine hedonic hunger prospectively to establish temporal relationships with weight gain and metabolic outcomes. Longitudinal studies could clarify whether hedonic hunger predicts future eating behaviors and health status among Indian youth. Additionally, intervention studies testing the effectiveness of hedonic hunger-targeted interventions in primary healthcare settings would provide valuable evidence for clinical practice. Research examining the neurobiological basis of hedonic hunger in Indian populations could enhance understanding of cultural variations in food reward processing. Finally, studies investigating the relationship between changing food environments in India and population-level patterns of hedonic hunger could inform policy approaches to obesity prevention.

## Conclusions

Hedonic hunger affects approximately one-quarter of youth attending primary healthcare services in Guntur, Andhra Pradesh, South India, with notable gender disparities; males and those from higher socioeconomic classes have a higher prevalence. The robust associations with modifiable lifestyle factors, particularly smoking, advertisement exposure, watching television while eating, and junk food consumption, suggest potential targets for effective interventions. These findings highlight the importance of incorporating the assessment of eating motivations into routine primary care and developing comprehensive approaches that address both media literacy and individual behavioral patterns. The results support the growing recognition that addressing the obesity epidemic requires attention to hedonic as well as homeostatic drivers of eating behavior. The predominance of hedonic hunger among males suggests the need for gender-specific approaches that account for different vulnerabilities and social contexts. Primary healthcare providers have a unique opportunity to identify youth at risk and implement early interventions targeting the psychological and environmental factors that may help reduce hedonic hunger.
